# Divergent evolution and purifying selection of the *flaA *gene sequences in *Aeromonas*

**DOI:** 10.1186/1745-6150-4-23

**Published:** 2009-07-21

**Authors:** Maribel Farfán, David Miñana-Galbis, M Carmen Fusté, J Gaspar Lorén

**Affiliations:** 1 Departament de Microbiologia i Parasitologia Sanitàries, Facultat de Farmàcia, Universitat de Barcelona, Av. Joan XXIII s/n, 08028 Barcelona, Spain

## Abstract

**Background:**

The bacterial flagellum is the most important organelle of motility in bacteria and plays a key role in many bacterial lifestyles, including virulence. The flagellum also provides a paradigm of how hierarchical gene regulation, intricate protein-protein interactions and controlled protein secretion can result in the assembly of a complex multi-protein structure tightly orchestrated in time and space. As if to stress its importance, plants and animals produce receptors specifically dedicated to the recognition of flagella. Aside from motility, the flagellum also moonlights as an adhesion and has been adapted by humans as a tool for peptide display. Flagellar sequence variation constitutes a marker with widespread potential uses for studies of population genetics and phylogeny of bacterial species.

**Results:**

We sequenced the complete flagellin gene *(flaA*) in 18 different species and subspecies of *Aeromonas*. Sequences ranged in size from 870 (*A. allosaccharophila*) to 921 nucleotides (*A. popoffii*). The multiple alignment displayed 924 sites, 66 of which presented alignment gaps. The phylogenetic tree revealed the existence of two groups of species exhibiting different FlaA flagellins (FlaA1 and FlaA2). Maximum likelihood models of codon substitution were used to analyze *flaA *sequences. Likelihood ratio tests suggested a low variation in selective pressure among lineages, with an ω ratio of less than 1 indicating the presence of purifying selection in almost all cases. Only one site under potential diversifying selection was identified (isoleucine in position 179). However, 17 amino acid positions were inferred as sites that are likely to be under positive selection using the branch-site model. Ancestral reconstruction revealed that these 17 amino acids were among the amino acid changes detected in the ancestral sequence.

**Conclusion:**

The models applied to our set of sequences allowed us to determine the possible evolutionary pathway followed by the *flaA *gene in *Aeromonas*, suggesting that this gene have probably been evolving independently in the two groups of *Aeromonas *species since the divergence of a distant common ancestor after one or several episodes of positive selection.

**Reviewers:**

This article was reviewed by Alexey Kondrashov, John Logsdon and Olivier Tenaillon (nominated by Laurence D Hurst).

## Background

The genus *Aeromonas *belonging to the *Gammaproteobacteria *includes a group of 21 Gram- negative bacterial species that can be isolated worldwide from a variety of environments. Although it is clear that members of this genus are primarily aquatic organisms, they can colonize other habitats and cause infections in invertebrates and vertebrates [1]. Recently, in their report of March 2006, the Office of Water of the Environmental Protection Agency of the USA (EPA) included some species of *Aeromonas *into a group of potential waterborne pathogens. Aeromonads are efficient colonizers of surfaces and are an important constituent of bacterial biofilms in both water distribution systems and food processing environments. Their polar flagellum contributes to biofilm formation and host colonization, as in the case of other bacterial genera such as *Campylobacter *and *Pseudomonas *[2]. Additionally, in recent studies the role of the polar flagellum of *Aeromonas *in enterocyte adherence has been found to be similar to those of other seafood pathogens such as *Vibrio *[3].

The bacterial flagellum is the most important organelle of motility in bacteria and plays a key role in many bacterial lifestyles, including virulence [4]. The flagellum also provides a paradigm of how hierarchical gene regulation, intricate protein-protein interactions and controlled protein secretion can result in the assembly of a complex multi-protein structure tightly orchestrated in time and space [5]. As if to stress its importance, plants and animals produce receptors specifically dedicated to the recognition of flagella [6,7]. Aside from motility, the flagellum also moonlights as an adhesion structure [3] and has been adapted by humans as a tool for peptide display [8].

Looking at the bacterial flagellum, instead of a simple model, there is an extensive variation in form and function. The most studied bacterial flagellar system is that of *Salmonella enterica *serovar Typhimurium, which exploits two different flagellins but never in the same filament, while in other cases, up to six different flagellins are incorporated into a single filament [9]. Flagellar filaments vary in their physical properties: some are rigid, others flexible, some are straight, others curly, some undergo post-transcriptional modifications such as glycosilation or methylation, others do not.

A comparison of the flagellins amino acid sequences (*fliC*, *flaA *and *flaB*) from many bacterial species has revealed a distinctive domain structure of the protein. The N- and C-terminal parts of the molecule, which are responsible for the secretion and polymerization, are conserved among species, whereas the central region, which produces the surface exposed antigenic part of the flagellar filament, is highly variable both within and among species. The N-terminal region comprises two domains, ND0 and ND1, with a highly conserved region between them, the spoke region (12 residues). The C-terminal region includes two domains, CD0 and CD1, with the corresponding counterpart to the spoke region, CS, between them [10]. The central variable region is less constrained, and includes sequences of variable length. Recent studies have shown that the N- and C-terminal conserved regions are recognized by the innate immune systems of mammals and plants [6]. In *Aeromonas*, two flagellin genes have been reported, *flaA *and *flaB*, which are present even in species described as non motile [11].

Natural selection has clearly rendered flagellar systems non-functional when they are no longer needed. Vestigial non-functional remnants of flagellar genes or regulons have been discovered in several bacterial species [12,13]. At least one degenerate flagellar system from *Brucella melitensis *still has a cryptic role in infection, even though it is no longer capable of mediating motility [14]. More recently, flagellar-related genes have been detected in the genome of *Myxococcus xanthus*, a soil bacteria that uses gliding rather than flagellar motility [5]. In addition, when examining homologies between flagellar and other bacterial proteins, it becomes clear that flagellar subunits share a common ancestry with components from other biological systems [15].

In this work we have determined the complete sequence of the *flaA *gene in 18 *Aeromonas *strains, including motile and non motile strains. Direct sequencing of the *flaA *gene has revealed the existence of two distinct FlaA flagellins (FlaA1 and FlaA2) that separate two groups of species (Group 1 and Group 2). The aim of the present study is to verify the role of the selective pressure acting on the *flaA *gene.

A comparison of synonymous (silent) and nonsynonymous (amino acid-changing) substitution rates in protein coding genes provides an important means for understanding molecular evolution. The nonsynonymous/synonymous rate ratio, ω (*d*_N_/*d*_S_), measures selective pressure at protein level. If nonsynonymous mutations are fixed at a lower rate than synonymous mutations, the ω ratio would be < 1 (purifying selection), while if both synonymous and nonsynonymous are fixed at the same rate, ω should be equal to 1 (neutrality). Finally, if nonsynonymous are higher than synonymous rates, ω should be >1 (positive selection).

To perform this study, we applied a maximum likelihood (ML) analysis based on models developed by Yang [16] to the 18 *flaA *gene sequence data. Researchers have begun to substitute the traditional null hypothesis testing approach for that of model selection, in which several competing hypotheses are simultaneously contrasted with the data. Models can be ranked and weighted, providing a quantitative measure of relative support for the competing hypothesis and in cases where models have similar levels of support from the data, model averaging can be used to make robust parameter estimates and predictions. This analysis is a valuable alternative especially when more than one hypothesis is plausible. Likelihood models developed by Yang [16] account for variable ω ratios among branches in the tree and can be used to test adaptative evolution along lineages. These models also allow the ω ratio to vary among amino acid sites and to identify critical amino acids under diversifying selection. In this work, we have applied the maximum likelihood models to our set of sequences in order to clarify the evolutionary pathway followed by the *flaA *gene in *Aeromonas*.

## Results

### Analysis of *flaA *gene

We have sequenced a total of 18 *flaA *genes corresponding to the full-length flagellin of different species of *Aeromonas *(Table 1). The results of the BLASTN search in the GenBank database verified that all sequences obtained were of the *Aeromonas *flagellin gene and showed high homology with the *flaA *gene. Sequences ranged in size from 870 (*A. allosaccharophila*) to 921 nucleotides (*A. popoffii*). The multiple alignment displayed 924 sites, 66 of which presented alignment gaps. Complete deletion of the sites with alignment gaps as well as the terminal stop codons resulted in an alignment of 855 nucleotides with 442 invariable sites (51.7%) and 413 variable sites (48.3%), 371 of which were parsimony informative. The identity of pairwise comparisons of the 18 sequences ranged from 69.2% (*A. sobria*/*A. bestiarum*) to 97.9% (*A. culicicola*/*A. veronii *bv. Sobria), with a mean of 77.4% ± 0.5.

**Table 1:** Origin of the *Aeromonas *strains used and characteristics of their *flaA *sequences

Species	Reference strain	Source/Country	*flaA *gene (bp)	G+C content (% mol)	GenBank accession n°.
*A. allosaccharophila*	CECT 4199^T^	Diseased eel/Spain	870	50.7	EU410305
*A. bestiarum*	CECT 4227^T^	Fish	912	55.0	EU410306
*A. bivalvium*	CECT 7113^T^	Cockle/Spain	918	58.3	EU410307
*A. caviae*	CECT 838^T^	Guinea pig	906	57.0	EU410308
*A. culicicola*	CIP 107763^T^	Mosquito/India	915	50.9	EU410309
*A. encheleia*	CECT 4342^T^	Healthy eel/Spain	900	56.1	EU410310
*A. eucrenophila*	CECT 4224^T^	Freshwater fish	900	57.7	EU410311
*A. hydrophila *subsp. *anaerogenes*	CECT 4221^T^	Used oil-emulsion	921	55.8	EU410312
*A. hydrophila *subsp. *dhakensis*	CECT 5744^T^	Human faeces/Bangladesh	912	57.8	EU410313
*A. hydrophila *subsp. *hydrophila*	CECT 839^T^	Milk	909	50.7	EU410314
*A. hydrophila *subsp. *ranae*	CIP 107985^T^	Frog/Thailand	912	56.2	EU410315
*A. jandaei*	CECT 4228^T^	Human faeces/USA	915	49.2	EU410316
*A. molluscorum*	CECT 5864^T^	Wedge-shell/Spain	906	57.6	EU410317
*A. popoffii*	LMG 17541^T^	Drinking water/Belgium	921	52.0	EU410318
*A. salmonicida *subsp. *salmonicida*	CECT 894^T^	Atlantic salmon/UK	918	52.4	EU410320
*A. salmonicida *subsp. *pectinolytica*	CECT 5752^T^	River water/Argentina	912	54.8	EU410321
*A. sobria*	CECT 4245^T^	Fish	903	51.1	EU410322
*A. veronii *bv. *Sobria*	CECT 4246^T^	Diseased frog	915	50.9	EU410323

Acronims: CECT, Spanish Type Culture Collection; CIP, Collection de l'Institut Pasteur; LMG, Belgian co-ordinated collections of microorganisms.

Abbreviations: ^T^, type strain; bp, base pairs; % mol, mole percent of G+C.

The average transition/transversion ratio was 1.0, two times the value expected with a random substitution distribution. The G + C content varied from 49.2% (*A. jandaei*) to 58.3% (*A. bivalvium*). The average nucleotide frequencies were 20.6%, 29.3%, 24.9% and 25.2% for T, C, A and G, respectively.

**Figure 1:**
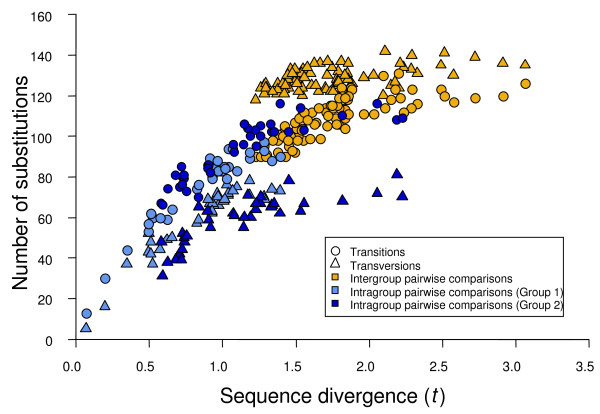
**Saturation plot of *flaA *sequences**. Number of transitions (circles) and transversions (triangles) from pairwise comparisons of 18 *Aeromonas *species and subspecies sequences plotted against their maximum likelihood distances calculated under the GTR + I + G substitution model (*t*).

Figure 1 depicts the saturation plot of *flaA *sequences using maximum likelihood distances. This graph strikingly shows a strong saturation of both transitional and transversional substitutions for sequence divergence values of up to 1.0, indicating the occurrence of multiple substitutions and possible homoplasy.

**Figure 2:**
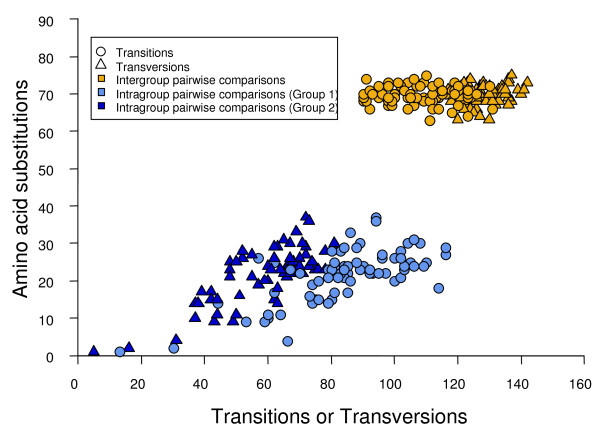
**Plot of amino acid substitutions against transitions and transversions for *flaA *sequences**. Number of amino acid substitutions plotted against transversions (triangles) and transitions (circles) from pairwise comparisons of sequences of 18 *Aeromonas *species and subspecies.

The number of amino acid substitutions of pairwise comparisons was plotted against the number of transition and transversion substitutions (Fig. 2). The graph demonstrates a strong saturation of both transitions and transversions in *flaA *sequences.

The mean number of transversions for pairwise comparisons between sequences pertaining to Group 1 and 2 was 128.27 ± 0.62 whereas the values for intragroups were: 60.06 ± 10.01 for Group 1 and 58.89 ± 9.81 for Group 2. In the case of transitions the intergroup value obtained was 108.53 ± 1.17, and in the case of the intragroup comparisons, the values obtained were 75.08 ± 3.13 and 92.89 ± 2.47 for Group 1 and 2, respectively.

Considering all these results, we decided to perform different genetic analyses in order to find the evolutionary models that could account for multiple substitutions and homoplasy as well as for the transition/transversion ratio and the different proportion of nucleotide base frequencies.

We compared 28 different models of nucleotide substitution: JC69 [17], K80 [18], F81 [19], F84 [20], HKY [21], N93 [22] and GTR [23,24]. In all cases models were modified by considering a proportion of invariant sites (option I) and/or assuming a gamma distribution of substitution rates among sites (option G). The General Time Reversible model (GTR) with a proportion of invariant sites of 0.432 and a gamma shape parameter α = 1.117 (GTR + I+ G) obtained the best Log likelihood scores. Hierarchical likelihood ratio tests and Akaike Information Criterion [25] both favoured the GTR + I + G model. The Akaike weight of this model, which can be interpreted as a probability, was 0.971 corroborating that the GTR + I + G is the most accurate model for the analysis of the observed data. This model implemented in the PHYML program was later used for inferring the phylogenetic tree of the 18 species and subspecies studied. However, identical or very similar topologies were obtained with other substitution models and clustering methods.

**Figure 3:**
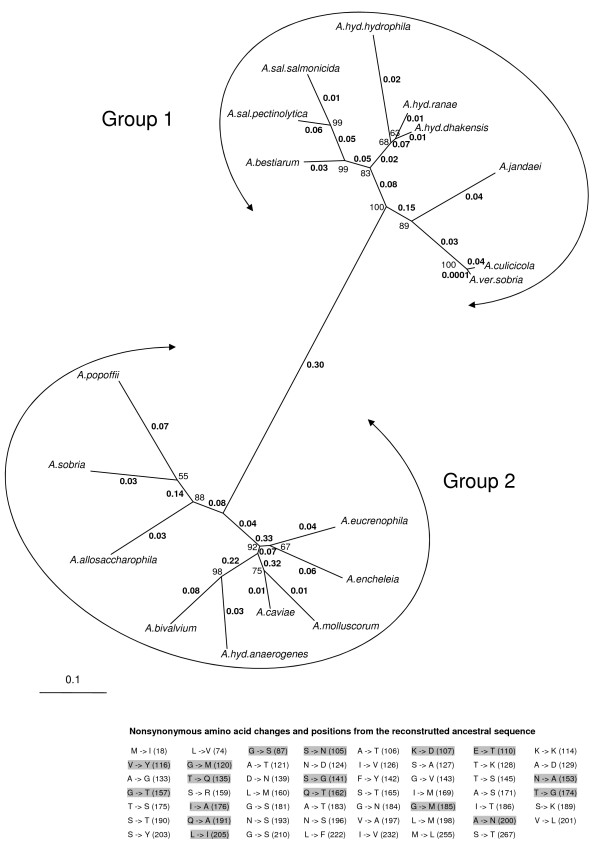
**The *flaA *gene maximum likelihood tree from 18 *Aeromonas *species**. Bootstrap values (1,000 replicates) are indicated in the nodes. Estimates of ω ratios, obtained under the "free-ratios" model, which assumes an independent ω ratio (*d*_N_/*d*_S_) for each branch in the tree, are shown in bold along branches. Ancestral sequences were reconstructed from Bayes Empirical Bayes analysis under a model that assumed a background ω ratio for branches of Group 1 and 2 (background branches) and another ω ratio for the branch connecting the two groups (foreground branch). Changes in the ancestral amino acids are indicated in the table (lower right position in the figure). Shadowed amino acid changes identify positions under positive selection, inferred from the M8 model (Table 6).

The tree shown in Figure 3 revealed two major clusters among the 18 *Aeromonas flaA *sequences. Group 1 includes the following strains: *A. bestiarum, A. culicicola, A. hydrophila *subsp. *dhakensis, A. hydrophila *subsp. *hydrophila, A. hydrophila *subsp. *ranae, A. jandaei, A. salmonicida *subsp. *pectinolytica, A. salmonicida *subsp. *salmonicida *and *A. veronii *bv. Sobria, and Group 2 contains *A. allosacharophila, A. bivalvium, A. caviae, A. encheleia, A. eucrenophila, A. hydrophila *subsp. *anaerogenes, A. molluscorum, A. popoffii *and *A. sobria*.

The mean intergroup number of pairwise nucleotide differences, 236.8 ± 1.4, was significantly higher than the mean intragroup pairwise differences: 135.1 ± 5.9 (Group 1) and 151 ± 4.1 (Group 2), (t test, *P *< 10^-15^). The differences between Group 1 and 2 were significant only at a level of 95% (t test, *P *= 0.03).

The amino acid alignment showed the existence of two different FlaA proteins among the *Aeromonas *species: FlaA1, which is present in *Aeromonas *species included in Group 1, and FlaA2, the flagellin exhibited by the species of *Aeromonas *belonging to Group 2. The comparative sequence analysis of these groups revealed the existence of amino acid residues fixed in different positions along the conserved regions of the protein, which varied between FlaA1 and FlaA2 flagellins. There were 14 of these residues in the N-terminal region (positions 18, 21, 74, 87, 105, 107, 114, 116, 121, 124, 126, 127, 133 and 142), 9 of which were in the ND1b domain (positions 105, 107, 114, 116, 121, 124, 126, 127 and 133), and 6 in the C-terminal region (positions 197, 198, 200, 205, 255 and 267). Only one of these fixed amino acid residues correspond to the central variable region (position 181). Furthermore, in the central region, all the sequences from species belonging to Group 1 exhibited a deletion of two amino acids (positions 164 and 165), which are not found in those of Group 2.

In order to clarify the role of selective pressures that operates in nucleotide diversity and sequence divergence of *flaA *in *Aeromonas *we performed a maximum likelihood analysis of *flaA *sequence data using the models developed by Nielsen and Yang [26] and Yang *et al*. [27], extending the model of codon substitution of Goldman and Yang [28] as implemented in the PAML4 package.

### Pairwise sequence comparisons of *d*_S _and *d*_N_

**Figure 4:**
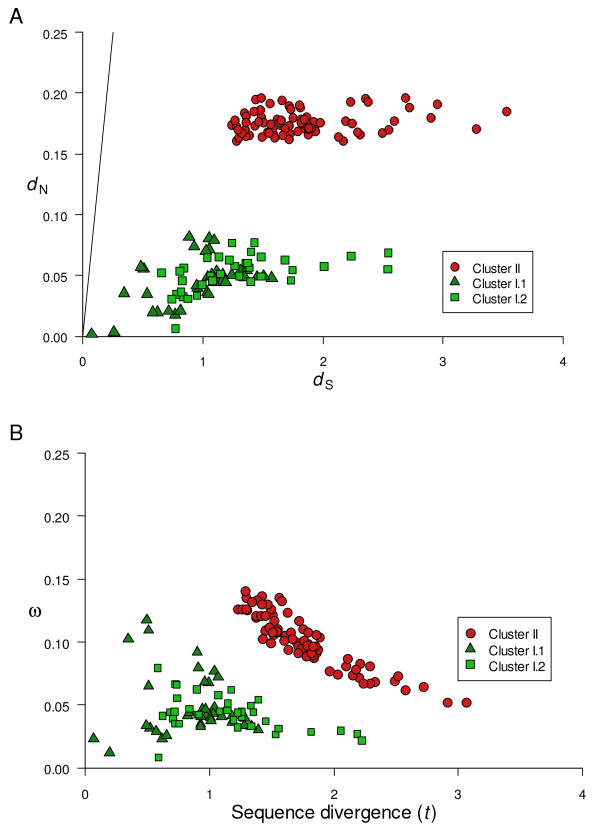
**Pairwise comparisons of *flaA *sequences from 18 *Aeromonas *species and subspecies**. Plot of maximum likelihood estimates of nonsynonymous (*d*_N_) against synonymous (*d*_S_) substitution rates (Fig. 4A), the straight line represents the neutral expectation (*d*_N _= *d*_S_). The ω ratio (*d*_N_/*d*_S_) plotted against the maximum likelihood estimates of sequence divergence (*t*), defined as the expected number of nucleotide substitutions per codon (Fig. 4B).

Pairwise comparisons of maximum likelihood estimates of synonymous (*d*_S_) and nonsynonymous (*d*_N_) substitution rates in the *flaA *sequences from the 18 *Aeromonas *strains are plotted in Figure 4A. Estimates obtained using the NG method [29] were almost identical and are not shown. All pairs showed *d*_N _values (range: 0.002 – 0.197) lower than *d*_S _(range: 0.074 – 3.529). The ratio *d*_N_/*d*_S _(ω) was thus lower than 1 in all the pairwise comparisons (range: 0.008 – 0.141) with a mean of 0.076 and a standard error of 0.003. We estimated by ML the *d*_S _and *d*_N _values with ω fixed to 1 and used a likelihood ratio test (LRT) to test the hypothesis that < 1, using ω = 1 as the null hypothesis. All pairwise comparisons showed *P *values < 10^-9^.

As shown in Figure 4A, the 153 pairwise comparisons fell in two discrete and strikingly delimitated clusters with *d*_N _ranging from 0.002 to 0.082 (Cluster I) and 0.161 to 0.197 (Cluster II). Cluster I represents the 36 intragroup pairwise comparisons corresponding to the species of Group 1 (Cluster I.1) and the 36 pairwise comparisons between species of Group 2 (Cluster I.2). Cluster II includes the 81 pairwise intergroup comparisons.

In Figure 4B, ω was plotted against sequence divergence (*t*), defined as the number of nucleotide substitutions per codon and was approximately 3.07 × (0.247 *d*_S _+ 0.753 *d*_N_), where 0.247 and 0.753 are the proportions of synonymous and nonsynonymous sites, respectively. In the case of pairs corresponding to Cluster II, there was a clear inverse relationship between ω and *t*, with a highly significant statistical correlation (*r *= -0.9032, *P *< 10^-15^). The correlation between ω and *t *for Cluster I.2 was also significant (*r *= -0.4507, *P *= 0.0058). However, in the case of Cluster I.1 the correlation between ω and *t *was not statistically significant (*r *= -0.1153, *P *= 0.5030).

### Variability of the ω ratio among codon sites

In order to verify the nature of the selective pressure acting on our alignment we used different codon-substitution models developed by Nielsen and Yang [26] and Yang *et al*. [27]. Standard codon models were fitted to the data set as implemented in the *codeml *program from the PAML package. Random-site models M0, M1a, M2a, M3, M7, M8 and M8a were fitted to our data; these models assume variation in ω among sites but not among lineages. The models used, parameter estimates, log-likelihood and AIC values of these models are shown in Table 2. Table 3 shows the results of the LRT tests for these models.

**Table 2:** Log-likelihood, AIC and parameter estimates under random-site models for *flaA *sequences

Model	*p*^a^	ω^b^	ℓ^c^	AIC^d^	κ^e^	Estimates of parameters	Positively selected sites
M0: one ratio	1	0.062 ± 0.000	-6830.350	13662.700	2.093	ω = 0.062	None

M1a: nearly neutral	2	0.114 ± 0.015	-6689.544	13383.089	2.192	*p*_0 _= 0.92 (*p*_1 _= 0.08) ω = 0.034, ω = 1	Not allowed

M2a: positive selection	4	0.114 ± 0.015	-6689.544	13387.092	2.192	*p*_0 _= 0.918, *p*_1 _= 0.082, (p_2 _= 0.000), ω_0 _= 0.034, ω_1 _= 1; ω_2 _= 58.267	179 I (at *P *= 0.747)

M3: discrete (K = 3)	5	0.071 ± 0.008	-6601.883	13213.766	2.085	*p*_0 _= 0.675, *p*_1 _= 0.254 (*p*_2 _= 0.071), ω_0 _= 0.003 ω_1 _= 0.122, ω_2 _= 0.546	None

M7: beta	2	0.071 ± 0.009	-6603.940	13211.879	2.087	*p *= 0.152, *q *= 1.683	Not allowed

M8: beta & ω > 1	4	0.072 ± 0.008	-6600.818	13209.635	2.082	*p*_0 _= 0.996, (*p*_1 _= 0.004) *p *= 0.160, *q *= 1.976 ω = **1.92**	**179 I **(at *P *= 0.967)

M8a: beta & ω_s _= 1	4	0.072 ± 0.008	-6601.490	13210.978	2.086	*p*_0 _= 0.991, (*p*_1 _= 0.009) *p *= 0.165, q = 2.190 ω = 1.0	Not allowed

^a ^*p*, number of free parameters for each model

^b ^ω, averaged ω ± standard error of *d*_N_/*d*_S _ratio over all sites in the *flaA *gene alignment

^c^ ℓ, log-likelihood value for each model

^d ^AIC, Akaike's Information Criterion value

^e^ κ, transition/transversion rate [27]

Values of ω > 1 and sites inferred to be under positive selection are in bold

**Table 3:** Likelihood Ratio Test statistics (LRT) for random-site models

Model 1^a^	Model 2^b^	2Δℓ^c^	df^d^	*P *– value
M3	M0	456.93	4	1.4 10^-97 ^***
M1a	M2a	0	2	1
M8	M7	6.24	2	0.044 *
M8a	M8	1.34	1	0.246

^a ^Alternative model; ^b ^null model; ^c ^2Δℓ = 2(ℓ_1 _- ℓ_0_), ^d^degrees of freedom (see Table 2).

*** significant at *P *< 0.001

* significant at *P *< 0.05

We applied the simplest of site-based models, M0 [30], which assumes an uniform ω ratio for all codons, to the data. The log likelihood for our sequences was ℓ = -6830.350, with an estimate of ω = 0.062, which can be interpreted as an average over all sites in the protein and all lineages in the tree. The low ω value obtained suggests a strong action of purifying selection in the evolution of *flaA *in the *Aeromonas *species studied.

Model M1a (nearly neutral), which hypothesizes a variable selective pressure among sites but not positive selection and M2a, which considers positive selection, fit our data better than the M0 model, with an ℓ = -6689.544 in both cases (Table 2). The LRT between M1a and M2a models allow the nearly neutral model to be rejected (Table 3). However, in the case of the M2a model, the proportion of sites with ω > 1 (p_2 _= 0) was null. Hence, no site was positively selected at a level of 95% and only an isoleucine in position 179 was identified as being under positive selection at a level of 75% (Table 2). These results also suggest the absence of positive selection among sites in *flaA *sequences.

In contrast, model M3 that assumes an unconstrained discrete distribution with three site classes (*p*_0_, *p*_1_, *p*_2_) and ω ratios (ω_0_, ω_1_, ω_2_) fits the data much better than M0 (ℓ = -6601.883); the value of the statistic test was 2Δℓ = 456.93, compared with the χ^2 ^distribution with df = 4, showing strong evidence against the null hypothesis (M0) in favour of the alternative (M3). However, the parameter estimates of the M3 model also suggest that all codons are under purifying selection with all sites with an ω < 0.6 (Table 2).

The most stringent test we carried out compared the M7 model, which assumes a beta distribution of ω over sites, with the M8, which adds an extra site class with a free ω ratio estimated from the data, allowing ω values greater than one (Table 2). Both models fitted the data better than M0 with values of ℓ = -6603.940 and -6600.818, respectively (Table 2). At a significance level of 95% we can reject the null hypothesis (M7 model) and accept the M8 model (Table 3), assuming the existence of ω values higher than 1, but at a level of 99% we should reject this hypothesis. In addition, the AIC value for the M8 model was only slightly lower (13209.635) than the value for the M7 model (13211.879).

**Figure 5:**
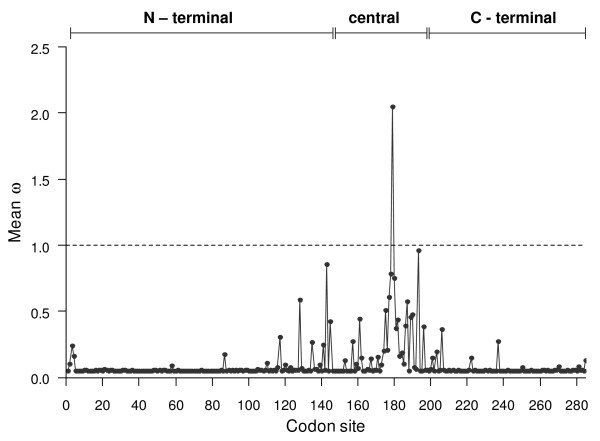
**Distribution of the posterior means of the ω ratio**. Posterior means of ω ratio under the M8 (beta & ω) model. In this model 10 equal-probability categories are used to approximate the beta distribution [27], giving 11 categories. The ordinate represents the average of ω over the 11 site classes, weighted by the posterior probabilities [30].

Even accepting M8 as the model that best fits our data, the parameter estimates showed that 99.6% of sites are under purifying selection and only one site (0.4%), the isoleucine in position 179, has an ω > 1 at a level of 95% (Table 2). Under this model, the ω average over the 285 codons was 0.073 with a variance of 0.024. Figure 5 shows the distribution of the posterior means of the ω ratio. The estimated distribution β (0.160, 1.976) has an extreme L shape, with an ω in the majority of sites close to 0. Assuming this model of ω distribution among sites, the ω mean would be 0.075 with a variance of 0.022, suggesting an extremely low probability that a site has an ω > 1. Estimates of the transition/transversion rate (κ) among the different models were very similar, with values ranging from 2.082 (M8 model) to 2.192 (M1a and M2a models) and a mean of 2.117 ± 0.02. These results confirm that purifying selection is the predominant force acting in the evolution of the *flaA *gene.

### Variability of the ω ratio among lineages

In order to identify those branches in the tree constructed from the *flaA *sequences with an ω ratio > 1 we applied the free-ratio models. The one ratio model (M0) assumes the same ω ratio for all lineages and when applied to our data involves a total of 44 parameters: branch lengths in the tree (33), nucleotide frequencies at the three codon positions (9), κ, and ω [31].

As shown in Table 2 the log-likelihood under this model was -6830.350, with ω = 0.062 as an average over all sites and lineages. We applied the free-ratio model, which assumes an independent ω ratio for each branch of the tree, to the same data set [31]. Estimates of the ω ratios are detailed in Figure 3. Considering the 33 branches of the tree, this model includes 32 additional parameters. The log-likelihood was ℓ_1 _= -6768.362. We performed an LRT using the M0 model as the null hypothesis. Comparison of 2Δℓ = 2(ℓ_1 _- ℓ_0_) = 123.98 with the χ^2^32 distribution suggests the rejection of the one-ratio model (*P *= 0.9 × 10^-12^). Although ω ratios varies among lineages (Fig. 3), the values of ω ratios corresponding to the different branches were significantly lower than one (t test, *P *= 2.2 × 10^-16^). The range was from 0.0001 to 0.3307 with a mean of 0.0845 and a standard error of 0.0156.

### Fixed-site models

The ω ratios inferred under the model M8 were scattered along the codon sites, with the highest values clustering in the region between codon 105 and 205 (Fig. 5). We applied the fixed-site models to our data in order to determine if the different sites along the sequences were under different selective constraints. We partitioned codon sites into three sections, 1: N-terminal (104 codons), 2: the central region (101 codons) and 3: the C-terminal (80 codons). The correspondence of these sections with the three regions of the flagellin is depicted in Figure 5.

The results obtained with the fixed site models are shown in Table 4. The statistics for the LRTs obtained by pairwise comparisons of these models are shown in Table 5. The simple model (model A) corresponds to random-site model M0, and assumes no site ω heterogeneity. The results suggest a strong action of purifying selection (ω = 0.062) along all the sites. Model E, which assumes different substitution rates (*r*s), transition/transversion ratio (κ), and a *d*_N_/*d*_S _ratio (ω), as well as different codon frequencies (πs), fitted the data significantly better than any of the simplest models (ℓ = -6580.030), and its Akaike weight was 0.999. However, parameter estimates were very similar under the different models (Table 4). It is noteworthy that the comparison between models D and C (2Δℓ = 0.23) leads to rejection of the model D, suggesting that the differences of κ between partitions were not significant. In order to test whether the ω ratio differed between partitions, we applied model D2, which was the same as model D except for fixing κ = 2.286. When comparing model D2 with model B (Table 5), the null hypothesis had to be rejected, indicating that although the ω ratio varies among partitions, it is lower than one (Table 4). Despite the heterogeneity of the substitution rates and *d*_N_/*d*_S _ratio in the *flaA *sequences, the results again confirm the hypothesis that purifying selection is the predominant force in the evolution of this gene.

**Table 4:** Log-likelihood values and parameter estimates under fixed-site models for *flaA *sequences

Model^a^	np^a^	ℓ	AIC	*r*s^b^	κ^b^	ω^b^
A (homogeneous model)	44	-6830.350	13748.700	1	2.093	0.062
B (different *r*s)	46	-6645.595	13383.190	*r*s_2 _= 4.473 *r*s_3 _= 1.614	2.286	0.049
C (different *r*s and πs^c^)	64	-6613.420	13354.840	*r*s_2 _= 5.595 *r*s_3 _= 1.608	2.262	0.046
D (different *r*s, κ, and ω)	50	-6613.537	13327.074	*r*s_2 _= 3.372 *r*s_3 _= 1.703	*κ*_1 _= 2.089 *κ*_2 _= 1.929 *κ*_3 _= 2.958	ω_1 _= 0.025 ω_2 _= 0.084 ω_3 _= 0.026
D2 (different *r*s and ω)^d^	47	-6617.228	13328.456	*r*s_2 _= 3.439 *r*s_3 _= 1.650	2.286	ω_1 _= 0.025 ω_2 _= 0.084 ω_3 _= 0.025
E (different *r*s, κ and ω, and different πs)	68	-6580.030	13296.060	rs_2 _= 3.944 *r*s_3 _= 1.688	*κ*_1 _= 1.929 *κ*_2 _= 1.929 *κ*_3 _= 3.005	ω_1 _= 0.023 ω_2 _= 0.083 ω_3 _= 0.025

The three partitions correspond to the 1: N-terminal extreme (104 codons), 2: the central region (101 codons) and 3: the C-terminal extreme (80 codons).

^a ^np, number of parameters including 33 branch lengths.

^b ^*r*s, nucleotide substitution rate. The subscript corresponds to the three partitions of *flaA *sequences (*r*s_1 _is 1 in all models and it is not shown); κ: transition/transversion ratio; ω: *d*_N_/*d*_S _ratio.

^c ^πs, codon frequencies (data not shown).

^d ^Model D but with κ fixed to 2.286.

**Table 5:** Likelihood Ratio Test statistics for fixed-site models

Model 1^a^	Model 2^b^	Parameter contrasted	2Δℓ^c^	df^d^	*P *value
B	A	different *r*s	369.51	2	5.8 10^-81 ^***
C	B	different πs	64.35	18	3.9 10^-7 ^***
D	C	different κ, ω	0.23	14	> 0.9999
D2	B	different ω	56.73	1	0.5 10^-13 ^***
E	D	different πs	67.01	18	1.4 10^-7 ^***

^a ^Alternative model; ^b ^null model; ^c ^2Δℓ = 2(ℓ_1 _- ℓ_0_), ^d ^degrees of freedom (see Table 4).

*** significant at *P *< 0.001

### Branch-site models

It is known that if a gene evolves under purifying selection most of the time but is occasionally subject to episodes of adaptive change, a comparison between two distant related sequences is unlikely to yield a *d*_N_/*d*_S _ratio significantly greater than one [32]. This could be the case of *flaA *sequences belonging to Group 1 and 2 (Fig. 3). Yang and Nielsen [33] implemented two models, A and B, which let the ω ratio vary both among sites and lineages. These models attempt to detect positive selection that affects only a few sites along a few lineages. Later modifications of these models [34,35] suggest that a likelihood ratio test, comparing the modified model A (still called branch-site model A) with the same model but with an ω ratio fixed to 1, appears to be a robust test for identification of positive selection sites. We applied this test to our data, labelling the branch that separates Group 1 from Group 2 as the foreground (Fig. 3). The results of this test are shown in Table 6.

**Table 6:** Parameter estimates for branch-site model A

	np	ℓ	Site class	Proportion	Background	Foreground	Selected sites under positive selection^c^
	
Model A^a^	4	-6631.240	0	0.779	0.027	0.027	**87G 105S ***107K*
			1	0.065	1	1	**110E 116V 120G**
			2a	0.144	0.027	39.95	**135T 141S ***153N*
			2b	0.012	1	39.95	*157G ***162Q 174K**
Model A with ω = 1^b^	3	-6638.340	0	0.712	0.026	0.026	*176T ***185G 191Q**
			1	0.060	1	1	**200A ***205L*
			2a	0.210	0.026	1	Not allowed
			2b	0.018	1	1	

^a ^Alternative model; ^b ^null model

^c ^Sites inferred under positive selection at the 99% level are in bold, and those at the 95% in italics

The LRT comparing the branch-site model with the null hypothesis model with ω = 1 gave a 2Δℓ = 14.20, and *P *= 0.0002, a significant value that indicated the existence of positive selection in this branch. We examined the Bayes Empirical Bayes (BEB) posterior probabilities to infer the sites that are likely to be under positive selection. The results of this test (Table 6) showed that 17 sites were identified as positively selected (*P *> 95%). We also applied an ML reconstruction of ancestral sequences using the codon model of Goldman and Yang [28] and Yang *et al*. [36]. It is noteworthy that all the 17 sites identified as positively selected among the amino acid changes are in the ancestral sequence revealed by the analysis (Fig. 3).

## Discussion

The flagellin gene is a widely applicable and useful genetic marker for studying variation within species of closely related bacteria [37]. Selander *et al*. have reported that the high level of variability and rapid evolution of flagellins provide a unique opportunity to test phylogenetic hypotheses [38]. In this work we studied 18 sequences of the *flaA *gene which codifies for the flagellar filament protein FlaA, obtained from different species and subspecies of *Aeromonas*.

The *flaA *gene presented a high degree of variability. When comparing the average identity of the sequences analyzed (77.44% ± 0.50), it was significantly lower than that determined for the 16S rRNA in the same group of strains (98.89% ± 0.04, *P *< 10^-86^) with sequences obtained from the databases. This value was even lower than that of the housekeeping gene malate deshydrogenase (*mdh*) (90.89% ± 0.25, *P *> 10^-64^), calculated from sequences obtained in our laboratory with the same strains (data not shown).

The *d*_N_/*d*_S _mean value of our sequences (0.076 ± 0.003) was similar to that determined by Jordan *et al*. [39] for 3106 orthologs (including essential and nonessential genes) in *E. coli *(0.081). However, it was much lower than that reported by Mamirova *et al*. [40] (0.462) for 22 *Gammaproteobacteria*. The *d*_N_/*d*_S _mean value obtained for the *flaA *sequences strongly suggests a predominant action of purifying selection. Nevertheless, if we focused our attention exclusively on the *d*_S _values, both the mean (1.494 ± 0.047) and the range (0.074–3.529) were notably high. These data agree with the saturation plot (Fig. 1), in which both transitions and transversions show a clear saturation for divergence values higher than 1.0.

The plot of amino acid substitutions against transitions and transversions (Fig. 2) clearly demonstrated a strong saturation in the *flaA *sequences when pairwise comparisons between the two groups (Group 1 and 2), were considered. Pearson's product moment correlation (*r*) between the number of transitions and the number of amino acid substitutions was 0.004 (F test, *P *= 0.972), which reveals the null influence of this type of mutations on the diversification of the two proteins. It is known that due to the structure of the genetic code, transversions produce more nonsynonymous changes and a higher proportion of radical amino acid changes, which involve an alteration of a certain physicochemical property [41]. Nevertheless, the correlation between the number of transitions and amino acid substitutions in pairwise comparisons between the *flaA *sequences corresponding to Group 1 and 2, although significant at a 95% level, was very low (*r *= 0.224; F test, *P *= 0.044), explaining only 5% of the variation. However, this correlation is significant when considering pairwise comparisons between the sequences. The product moment correlation values for transitions and transversions in Group 1 were *r *= 0.792 (F test, *P *< 10^-8^) and *r *= 0.800 (F test, *P *< 10^-8^), respectively, and in Group 2 were *r *= 0.602(F test, *P *= 0.0001) and *r *= 0.761 (F test, *P *< 10^-7^), respectively.

Most of the models applied to the *flaA *sequences determined that nearly all the sites are under purifying selection with only one site, the isoleucine in position 179, having an ω > 1 at a level of 95% (Tables 2, 4 and 6). This is not surprising since this amino acid is located in the central region of the flagellin, a surface exposed domain that is free to vary far more widely. Indeed, in other bacterial species the central region is considered to be under neutral or positive selection pressure [38]. Nevertheless, the branch-site model suggests episodes of positive selection in the separation of the two flagellins in *Aeromonas *species. The branch-site models attempt to detect positive selection that affects only a few sites along a few lineages [34,35]. In the case of branch-site model A, all the sites positively selected are included in those identified as ancestral (Fig. 3, Table 6). Seven of the 17 sites identified as positively selected correspond to fixed amino acids that are identical for all the species included in a group but different between groups. The remaining sites correspond to positions with fixed amino acids in one group but not in the other. In addition, twelve of the positively selected 17 sites are radical amino acid substitutions. In conclusion, all the positions identified as positively selected contributed to the diversification of the two flagellins detected in *Aeromonas*, FlaA1 and FlaA2.

These results can be explained considering the possible existence of two *flaA *genes (*flaA1 *and *flaA2*) among the *Aeromonas *species studied. These genes would have been diverging over a long period of time accumulating all the possible substitutions leading to amino acid changes allowed by the functional constraints of the flagellins. The results obtained with the codon models that account for selection on different codon sites or within specific lineages (Tables 2 and 4) suggested that both transitions and transversions would be submitted to a strong purifying selection.

In the analysis described in this paper, alignment gaps in the variable region of the flagellin were removed. It is known that in many bacterial species, the central region of the genes that codify the flagellin (*flaA*, *flaB*, *fliC*) is polymorphic and highly diverse with numerous gaps [10]. In order to determine the effect of alignment gaps, we also analyzed our sequences with the 66 alignment gaps included and treated them as ambiguity data [42,43]. The sequences then contained 307 codons (921 nucleotides). When comparing the sequences, with and without gaps using the one ratio estimate model, the values obtained were very similar, 0.078 and 0.062, respectively (Table 2). In addition, both values were clearly lower than one. When we determined the parameter estimates using the M8 model (beta & ω > 1) the values obtained (*p*_0 _= 0.982, *p*_1 _= 0.018, *p *= 0.160, *q *= 1.670) were similar to those shown in Table 2. The only site identified as being under positive selection was again the isoleucine, now in position 182 because of the gaps included in the analysis. We also carried out the analysis of the sequences using the branch-site model A (Table 4) to corroborate if the presence of alignment gaps influenced the detection and identification of sites under positive selection. The results confirm that the sites identified as being under positive selection were identical to those previously identified when we conducted the analysis excluding the gaps.

These results demonstrated that although alignment gaps in the sequence analyses have a certain impact on the estimates of model parameters; they do not seem to influence the identification of sites under positive selection. However, insertions and deletions can play an important role in the divergence of both flagellins (e.g. the indel of two amino acids that appears in all the species of Group 1, positions 164 and 165).

## Conclusion

In conclusion, the maximum likelihood models applied to our set of sequences allowed us to determine the possible evolutionary pathway followed by the *flaA *gene in *Aeromonas*. The results indicated that this gene has probably been evolving independently in the two groups of *Aeromonas *since the divergence of a distant common ancestor after one or several episodes of positive selection in the branch that separates these groups. The saturation of nonsynonymous changes determined in the *flaA *sequences revealed that the flagellins that define those groups of species have accumulated all the amino acid changes allowed by the restriction structural constraints of the protein.

## Methods

### Bacterial strains and DNA isolation

A total of 18 *Aeromonas *strains were analyzed including type and reference strains of the genera (Table 1). All strains were grown aerobically on Tryptone soy agar (TSA) at 30°C. Isolates were stored in TSB containing 20% glycerol at -40°C. Genomic DNA was extracted with the REALPURE^® ^Genomic DNA extraction kit (REAL RBMEG03, Durviz, Spain), and stored at 20°C until use.

### Primer design

Specific primers were designed from published polar flagella operons of some *Aeromonas *species [2,11,44] and the complete genome sequence of *A. salmonicida *subsp. *salmonicida *A449 (GenBank accession number CP000644). For PCR amplications, three sets of primers were designed to amplify the complete *flaA *gene and its flanking regions (*yadS *and *flaB *genes). The sense primers fla1 (5'-GCTGATGTAAACAATCTGCT-3') and fla5 (5'-GCTTAGGAGAATGGTTATG-3') are located at 437 nt and 16 nt, respectively, upstream of the translational start site of the *flaA *gene. The antisense primers fla2 (5'-GCGTTCAGTGATGAAGTATT-3') and fla2A (5'-CACCCCNTTGTTCCATCT-3') are located at 1541 nt and 977 nt, respectively, downstream of the initiator codon. A set of internal primers was used for sequencing PCR products: fla1A-2 (sense, 5'-ATCAACAGCGCNAANGA-3'), fla12 (sense, 5'-TCCAACCGTCTGACCTC-3'), fla10 (sense, 5'-GCCATGGATGAAGTGAC-3'), fla11 (sense, 5'-CANGTNGGNGCNGANGC-3') and fla7 (antisense, 5'- CGGTTNTGNACCGCACC-3'), which were located at base positions 112, 151, 241, 439 and 722, respectively, downstream of the translational start. Primer3 software was used to design PCR and sequencing primers . The oligonucleotides were synthesized by Isogen Life Science (Maarssen, The Netherlands).

### PCR amplification and DNA sequencing

PCR reactions were carried out in a 50 μl volume, containing 0.5–10 μl of genomic DNA as template, 1× PCR buffer I (10× PCR buffer I: 500 mM KCl, 15 mM MgCl_2_, 100 mM Tris-HCl [pH 8.3]), 2 mM MgCl_2_, 0.2 mM each dNTP, 10 pmol each primer and 1 U of AmpliTaq Gold DNA polymerase (Applied Biosystems). Amplifications were performed in an Applied Biosystems 2720 thermal cycler using the following program: initial denaturation at 94°C for 5 min, followed by 35 cycles of denaturation at 94°C for 45 s, annealing at 45–50°C for 1 min and elongation at 72°C for 1.30 min, and a final extension at 72°C for 10 min. PCR products were resolved by electrophoresis, and amplicons were purified with a MSB^® ^Spin PCRapace kit (Invitek) or from agarose gel with a QIAquick^® ^Gel Extraction kit (Qiagen). Purified PCR products were directly sequenced on both strands using either the PCR primers or internal primers. Sequencing reactions were performed with an ABI PRISM^® ^BigDye^® ^Terminator v3·1 Cycle Sequencing kit (Applied Biosystems) and analyzed on an ABI PRISM 3700 DNA sequencer (Applied Biosystems) by the Genomics Unit of the Scientific and Technical Services of the University of Barcelona (SCT-UB).

### Data analyses

The nucleotide sequences obtained were examined for open reading frames (ORFs) with the ORF finder program and were also compared to the GenBank database using a BLASTN analysis, both available at the NCBI . Sequence data were translated, aligned using ClustalX [45] according to the system default parameters, and back translated to obtain the nucleotide alignments. The multiple alignment contained 66 sites with alignment gaps. These sites and the termination codons were removed, giving sequences of 855 bp length for all strains (285 codons). All subsequent analyses were performed on this set of aligned DNA sequences.

The PHYML package [46] was used for tree reconstructions in combination with the most likely model of nucleotide substitution identified by two hierarchical tests, the likelihood ratio test (LRT) and Akaike's information criterion (AIC) [25,47]. Model selection and tree statistics were done using the APE package [48] and R language [49]. The DnaSP software [50] was used to obtain the DNA polymorphism data. The identity of the sequences, the number of transitions and transversions and the amino acid substitutions were calculated by using the MEGA 4 program [51].

The synonymous substitutions rate (synonymous substitutions per synonymous site *d*_S_), nonsynonymous substitutions rate (nonsynonymous substitutions per nonsynonymous site, *d*_N_) and *d*_N_/*d*_S _ratio (ω) were calculated using the Nei-Gojobori (NG) method [29] and the codon-based model of Goldman and Yang [28] as implemented in the *codeml *program of the PAML 4 package [16]. The latter is a maximum likelihood (ML) method based on an explicit model of codon substitution, which accounts for the transition/transversion rate (κ), ω, and the base frequencies at the three codon positions.

Standard codon models were fitted to the data set with the PAML software, and likelihood ratio tests (LRT) were used to determine the relative fit of the hierarchically nested models. Likelihood ratio test statistic 2Δℓ = 2(ℓ_1 _- ℓ_0_), where ℓ_1 _represents the log-likelihood of the model corresponding to the alternative hypothesis and ℓ_0 _is the log-likelihood corresponding to the model used as null hypothesis, were compared with a chi-squared distribution in which the difference between the number of parameters of both models gives the degrees of freedom (χ^2^_df_) [30].

Models tested included those that account for selection in different codon sites, within specific lineages, or a combination of selection in different sites within specific lineages. Parameters and other details involved in these models are explicit in the Results section. For all models the equilibrium codon frequencies were estimated from the products of the average observed nucleotide frequencies in the three codon positions (option F3 × 4 in the PAML package). To verify convergence all PAML analyses were run at least three times from distinct starting values of parameters and seed used.

Other statistical analyses and graphics were done in the R language and Microsoft Excel. Unless otherwise indicated, uncertainty is expressed as the standard error.

### Nucleotide sequence accession numbers

The nucleotide sequences determined in this study have been deposited in the GenBank database under accession numbers EU410305 to EU410318 and EU410320 to EU410323.

## Competing interests

The authors declare that they have no competing interests.

## Authors' contributions

MF and MCF designed and performed the experiments. DMG contributed to the experiments and preliminary data analysis. MCF, MF and DMG contributed to writing the manuscript. JGL ran the PAML package, interpreted the results and supervised the manuscript. All authors read and approved the final manuscript.

## Reviewers' Comments

Reviewer 1

Dr Alexey Kondrashov, University of Michigan, Life Sciences Institute, Michigan, USA This reviewer provided no comments for publication.

Reviewer 2

Dr John Logsdon, Department of Biology, University of Iowa, Iowa City, USA

### Reviewer comments

This paper reports the isolation and analysis of flagellin gene sequences from a diverse collection of 18 *Aeromonas *species and subspecies. Genes were isolated by PCR and the resulting products were sequenced directly. By this method, each species yielded a single flagellin gene sequence. The authors use detailed molecular evolutionary analyses to assess the pattern and strength of selection on these genes.

The phylogenetic analysis showed two distinct groupings of these genes that the authors term FlaA1 and FlaA2. Even though it is well-known that *Aeromonas *species generally contain two tandemly-linked flagellin genes (FlaA and FlaB), the authors seem to have missed the most logical interpretation that their so-called FlaA1 and FlaA2 are actually FlaA and FlaB (respectively, or vice versa). Unfortunately, the authors seemed to have missed this obvious conclusion, made even stronger by the fact that the underlying *Aeromonas *species phylogeny (as recently published by Saavedra *et al*., 2006, IJSEM 56: 2481) is not at all congruent with the authors' flagellin gene phylogeny shown in their Figure 3.

*Author's response:*

Regarding the main objection you pointed out in your revision, the misinterpretation of the two flagellin genes, we are aware that the flagellin operon of *Aeromonas *is composed of the *flaA *and *flaB *genes as cited in the manuscript (background: paragraph 4).

Concerning the phylogeny of *Aeromonas *published by Saavedra *et al*., the incongruences are not surprising because the work of these authors is based on the partial sequences of two housekeeping genes (*gyrB *and *rpoD*) that are supposed to be selectively neutral, while we have analyzed sequences from a gene that codifies an external protein that is subjected to high selective pressure. Despite the above, our intention was not to construct a phylogeny of *Aeromonas *based on the sequences of *flaA *gene.

*Reviewer comments:*

This appears to be a classic case of not discerning between paralogs and orthologs in a phylogenetic analysis caused by the arbitrary selection of one paralog or the other from a given genome. It is clear that the two groups of flagellar genes (which I interpret to be FlaA and FlaB) are paralogs that diverged early in *Aeromonas *evolution. The fact that each *Aeromonas *species gave only one sequence (either FlaA1 or FlaA2, according to the authors' naming) could be simply due to the direct sequencing of amplicons. Presumably, any mixed amplicons would yield poor sequence, since paralogs within species are ~80 identical; but if clones from those presumed-mixed amplicons were prepared and sequenced, then two distinct versions would be predicted.

The serious issue in not distinguishing between paralogous flagellin genes (i.e., FlaA and FlaB) undermines the entire analysis of this paper and its interpretation. In my view this fundamental flaw renders the paper unacceptable for publication in Biology Direct at this time. However, the details of the selection analyses of the sequences per se appear to be sound, even if the evolutionary context necessary for appropriate interpretation is indeed incorrect. Thus, with a careful re-framing of the analysis in a manner that determines and then clarifies the appropriate evolutionary history of these genes in *Aeromonas*, the paper would likely be appropriate for publication in Biology Direct.

*Author's response:*

The presence of two flagellin genes located genetically in tandem in *Aeromonas *has been taken into account when we designed the primers used for the PCR amplification and direct sequencing of the full length sequences of the *flaA *gene (methods section). The PCR primers used were designed from the two genes flanking *flaA *in *Aeromonas*, *yadS *(forward) and *flaB *(reverse). With these primers we obtained a sequence that includes the full length *flaA *gene plus a region upstream that contains part of *yadS *and another downstream that includes part of the *flaB *gene. As a consequence it is not possible to mistake the amplified region and we are sure in all cases we amplified *flaA*.

In addition, we conducted a BLASTN analysis with the sequences obtained from our strains and in all cases without exceptions, the sequences showed a high homology with other *Aeromonas flaA *sequences available in the GenBank as we explained in the manuscript (results: paragraph 1).

Finally, in order to confirm that our sequences correspond to the *flaA *gene we constructed a new maximum likelihood tree (additional file 1), in which we have incorporated four *flaB Aeromonas *sequences from the GenBank database. As you can see, *flaB *sequences clustered in a second major branch in both groups (Group 1 and Group 2), showing that probably they could also be two alleles of *flaB *genes in *Aeromonas *as we observed in the case of *flaA*, or even two different flagellin operons.

Reviewer 3

Dr Olivier Tenaillon, INSERM U722, Faculté de Médecine Xavier Bichat, Paris, France (nominated by Dr Laurence D Hurst)

### Reviewer comments

The present article provides an analysis of the flagellin gene *flaA *in the opportunistic pathogen species complex of *Aeromonas*. As flagella is exposed it has been shown to be under diversifying selection in several species, it appear to be a good candidate to study traces of positive selection with PAML. Yet in the present study most signal are compatible with stabilizing selection rather than diversifying selection.

I think to be more relevant, the analysis should be coupled with a phylogenetic analysis of a gene subject to less selective pressure such as *mdh *that the author mention they have sequenced too. This will for instance give more power to the analysis of the diversification of *flaA *in two groups. Are the species showing the same pattern or is it specific to *flaA *gene? If the diversification is specific this will comfort the idea that positive selection has been at work.

*Author's response:*

As the referee suggest, we have included an additional file (additional file 2), which shows the phylogenies inferred from *mdh *and *flaA *gene sequences from different *Aeromonas s*pecies and subspecies. The trees depict distinct phylogenies, *mdh *being much more in accordance with other housekeeping gene phylogenies for *Aeromonas*, and confirming that the two groups of strains obtained in the *flaA *tree were a consequence of the divergence of the two alleles of *flaA*, suggesting an early episode of positive selection in their origin.

### Reviewer comments

However one possible explanation is also that a duplication occurred and that a single copy get conserved or sequenced in each subgroup. Could this be simply rejected?

*Author's response:*

Following the Occham's razor principle, the simplest and most probable hypothesis is that in prokaryotes, after a duplication, both copies of the gene are conserved, usually in tandem or, less frequently, in a distant region of the chromosome. Concerning the sequencing of a single copy in each subgroup, this fact should be rejected, considering that, as we mentioned above in the answer to Dr Logsdon, the PCR primers used were designed from the two genes flanking *flaA *in *Aeromonas*, *yadS *(forward) and *flaB *(reverse). With these primers we obtained a sequence that includes the full length *flaA *gene plus a region upstream that contains part of *yadS *and another downstream that includes part of the *flaB *gene. As a consequence it is not possible to mistake the amplified region and we are sure in all cases we amplified *flaA*.

### Reviewer comments

The strong saturation observed could limit the usage of the PAML like software, it could be appropriate to split the dataset in the two groups to see if any more signal would emerge. Also along those lines, the estimation of omega along the sequence as shown in the figure 5 reveals a very contrasted pattern between 0–120, 120–210, and 210 till the end of the protein. Analyzing those regions separately could also give increased power. Indeed branch site models should in theory do the job of separating branches and sites but as they compare to other sites and branches, having large heterogeneity might affect the power to detect selection.

*Author's response:*

Firstly, we are not sure about the possibility of splitting the dataset in two groups because of the low number of sequences in each of them (nine). Anyway, we have conducted the PAML analysis considering these two groups as you suggest, and the results obtained are not conclusive. Estimation of omega along different parts of the sequence protein shows that although the central region is less constrained (with omega values 0.0787) than the other two regions N- and C-terminal (with omega values 0.032 and 0.021, respectively), all these values are clearly lower than one, and do not modify the results.

*Reviewer comments:*

I do not understand the information brought by the assertion «site under positive selection were found in the ancestral sequence», also in the conclusion the idea that all possible amino acid have been visited appear incompatible with *d*_N_/*d*_S _less than one and strong purifying selection. If no strong positive selection is observed in the recent diversification of the *flaA *gene could the biological implication be discussed as *Aeromonas *are mainly opportunistic pathogens.

*Author's response:*

We mean that the 17 sites identified as positively selected (*P *> 95%), when we applied the branch-site model A to our sequences (Table 6), were all of them are in the reconstructed ancestral sequence from which Group 1 and 2 diverged. Moreover, as that possibly happened a long time ago and since then both groups of sequences have evolved under purifying selection, the rate ratio of *d*_N_/*d*_S _is less than one.

## Supplementary Material

Additional file 1 **Unrooted maximum likelihood tree of flagellins of *Aeromonas***. The tree was inferred by the maximum likelihood method, using the GTR+I+G model of nucleotide substitution, for comparison of 18 *flaA *sequences obtained in this study and 4 *flaB *sequences of *Aeromonas *available in the GenBank database under the accession numbers AF198617 (*A. caviae*), AY839592 (*A. punctata*), NC_009348 (*A. salmonicida *subsp. *salmonicida*) and DQ119104 (*A. hydrophila*). Bootstrap values greater than 50% from 500 resamplings are indicated at each node. The scale bar represents 0.1 nucleotide substitutions per position. The *flaB *sequences of *Aeromonas *are indicated in bold. Click here for file

Additional file 2 **A comparison of *flaA *and *mdh *gene trees**. Neighbour-joining trees of *flaA *(left) and *mdh *(right) gene sequences were constructed based on Kimura-2-parameter (K2P) distances using the MEGA software. Bootstrap values over 50% from 1000 resamplings are shown for each node. The scale bar at the bottom of each tree represents the K2P genetic distance. Click here for file
