# Supplementary material for: Divergent evolution and purifying selection of the flaA gene sequences in Aeromonas
Source: Biol Direct. 2009 Jul 21;4:23. doi: 10.1186/1745-6150-4-23 (PMC2724415; doi:10.1186/1745-6150-4-23)
Supplement: Additional file 1 — Unrooted maximum likelihood tree of flagellins of Aeromonas. The tree was inferred by the maximum likelihood method, using the GTR+I+G model of nucleotide substitution, for comparison of 18 flaA sequences obtained in this study and 4 flaB sequences of Aeromonas available in the GenBank database under the accession numbers AF198617 (A. caviae), AY839592 (A. punctata), NC_009348 (A. salmonicida subsp. salmonicida) and DQ119104 (A. hydrophila). Bootstrap values greater than 50% from 500 resamplings are indicated at each node. The scale bar represents 0.1 nucleotide substitutions per position. The flaB sequences of Aeromonas are indicated in bold. [file 1745-6150-4-23-S1.ppt]

## Slide 1
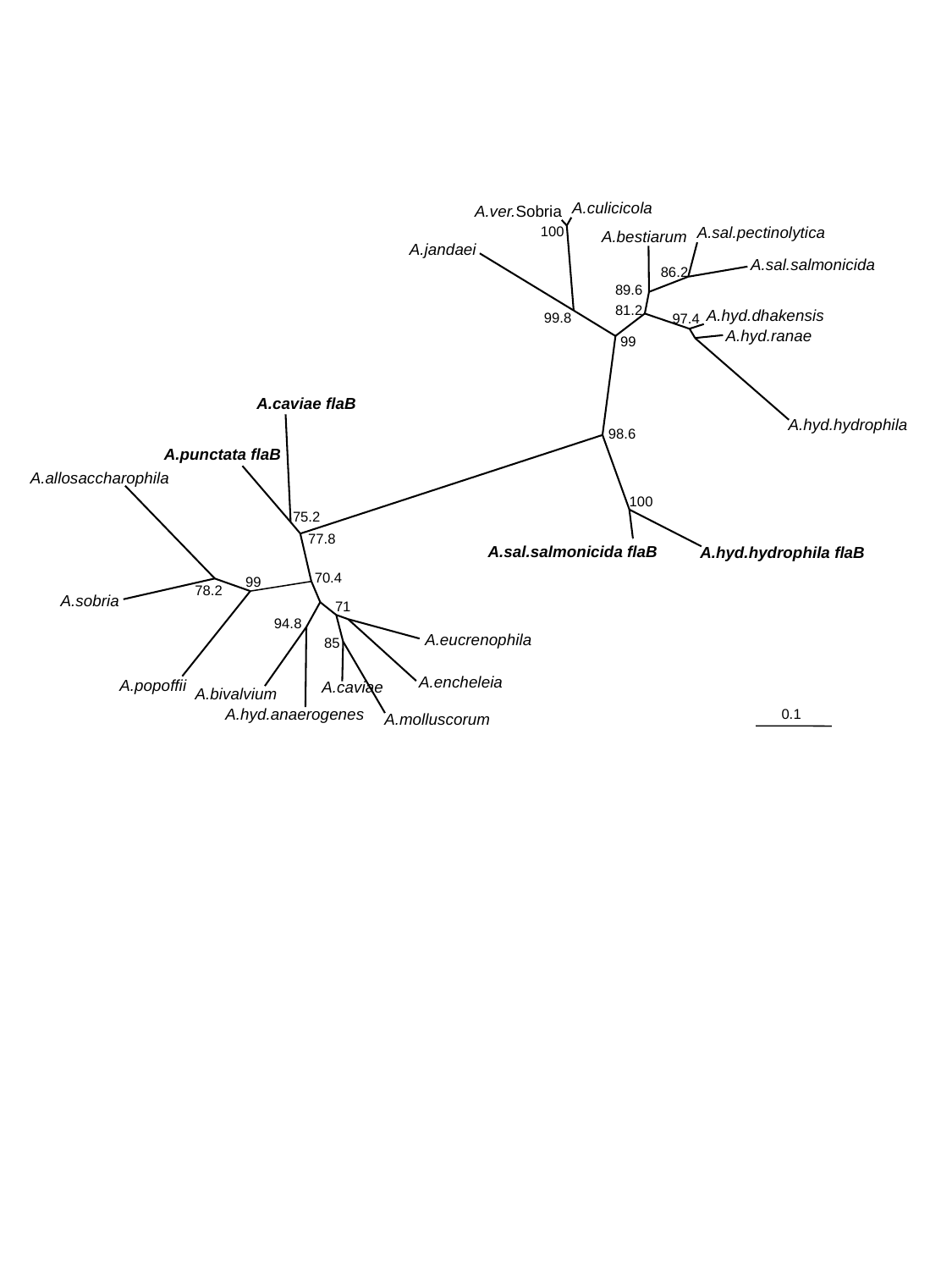

A.culicicola
A.ver.Sobria
A.sal.pectinolytica
100
A.bestiarum
A.jandaei
A.sal.salmonicida
86.2
89.6
81.2
A.hyd.dhakensis
99.8
97.4
A.hyd.ranae
99
A.caviae flaB
A.hyd.hydrophila
98.6
A.punctata flaB
A.allosaccharophila
100
75.2
77.8
A.sal.salmonicida flaB
A.hyd.hydrophila flaB
70.4
99
78.2
A.sobria
71
94.8
A.eucrenophila
85
A.encheleia
A.popoffii
A.caviae
A.bivalvium
A.hyd.anaerogenes
0.1
A.molluscorum
