# Supplementary material for: Divergent evolution and purifying selection of the flaA gene sequences in Aeromonas
Source: Biol Direct. 2009 Jul 21;4:23. doi: 10.1186/1745-6150-4-23 (PMC2724415; doi:10.1186/1745-6150-4-23)
Supplement: Additional file 2 — A comparison of flaA and mdh gene trees. Neighbour-joining trees of flaA (left) and mdh (right) gene sequences were constructed based on Kimura-2-parameter (K2P) distances using the MEGA software. Bootstrap values over 50% from 1000 resamplings are shown for each node. The scale bar at the bottom of each tree represents the K2P genetic distance. [file 1745-6150-4-23-S2.ppt]

## Slide 1
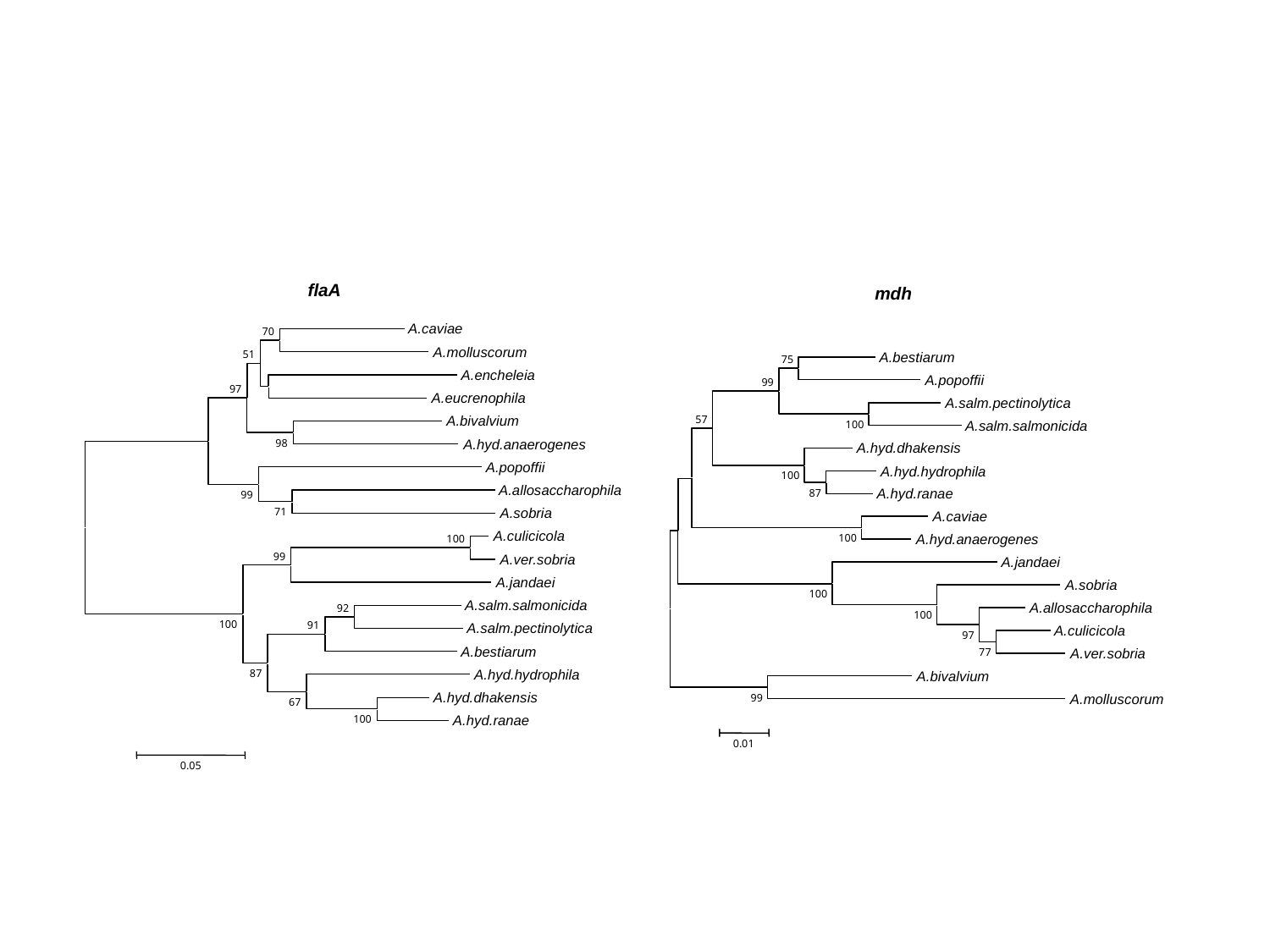

flaA
 A.caviae
70
 A.molluscorum
51
 A.encheleia
97
 A.eucrenophila
 A.bivalvium
 A.hyd.anaerogenes
98
 A.popoffii
 A.allosaccharophila
99
 A.sobria
71
 A.culicicola
100
99
 A.ver.sobria
 A.jandaei
 A.salm.salmonicida
92
100
91
 A.salm.pectinolytica
 A.bestiarum
 A.hyd.hydrophila
87
 A.hyd.dhakensis
67
 A.hyd.ranae
100
0.05
mdh
 A.bestiarum
75
 A.popoffii
99
 A.salm.pectinolytica
57
 A.salm.salmonicida
100
 A.hyd.dhakensis
 A.hyd.hydrophila
100
 A.hyd.ranae
87
 A.caviae
 A.hyd.anaerogenes
100
 A.jandaei
 A.sobria
100
 A.allosaccharophila
100
 A.culicicola
97
 A.ver.sobria
77
 A.bivalvium
 A.molluscorum
99
0.01
